# Prediction of survival time after terminal extubation: the balance between critical care unit utilization and hospice medicine in the COVID-19 pandemic era

**DOI:** 10.1186/s40001-022-00972-w

**Published:** 2023-01-11

**Authors:** Yun-Cong Zheng, Yen-Min Huang, Pin-Yuan Chen, Hsiao-Yean Chiu, Huang-Pin Wu, Chien-Ming Chu, Wei-Siang Chen, Yu-Cheng Kao, Ching-Fang Lai, Ning-Yi Shih, Chien-Hong Lai

**Affiliations:** 1 Departments of Neurosurgery, Chang Gung Memorial Hospital, Keelung and Linkou & Chang Gung University, Taoyuan, Taiwan; 2 Department of Biomedical Engineering, National Taiwan University, Taipei, Taiwan; 3 Division of Hematology-Oncology, Department of Internal Medicine, Chang Gung Memorial Hospital, No. 222, Maijin Rd., Anle Dist., Keelung, 204 Taiwan; 4 Institute of Medicine, Chung Shan Medical University, Taichung, Taiwan; 5 School of Nursing, College of Nursing, Taipei Medical University, Taipei, Taiwan; 6 Research Center of Sleep Medicine, College of Medicine, Taipei Medical University, Taipei, Taiwan; 7 Department of Nursing, Taipei Medical University Hospital, Taipei, Taiwan; 8 Division of Pulmonary, Critical Care and Sleep Medicine, Chang Gung Memorial Hospital, Keelung, 20401 Taiwan; 9 College of Medicine, Chang Gung University, Taoyuan, 33302 Taiwan; 10 Division of Cardiology Section, Internal Medicine, Chang Gung Memorial Hospital, Keelung & Chang Gung University, Taoyuan, Taiwan; 11 Department of Social Services, Chang Gung Memorial Hospital, Keelung, Taiwan

**Keywords:** Terminal extubation, APACHE II score, Hospice medicine, SpO2, Intensive care unit, COVID-19

## Abstract

**Background:**

We established 1-h and 1-day survival models after terminal extubation to optimize ventilator use and achieve a balance between critical care for COVID-19 and hospice medicine.

**Methods:**

Data were obtained from patients with end-of-life status at terminal extubation from 2015 to 2020. The associations between APACHE II scores and parameters with survival time were analyzed. Parameters with a *p*-value ≤ 0.2 in univariate analysis were included in multivariate models. Cox proportional hazards regression analysis was used for the multivariate analysis of survival time at 1 h and 1 day.

**Results:**

Of the 140 enrolled patients, 76 (54.3%) died within 1 h and 35 (25%) survived beyond 24 h. No spontaneous breathing trial (SBT) within the past 24 h, minute ventilation (MV) ≥ 12 L/min, and APACHE II score ≥ 25 were associated with shorter survival in the 1 h regression model. Lower MV, SpO2 ≥ 96% and SBT were related to longer survival in the 1-day model. Hospice medications did not influence survival time.

**Conclusion:**

An APACHE II score of ≥ 25 at 1 h and SpO2 ≥ 96% at 1 day were strong predictors of disposition of patients to intensivists. These factors can help to objectively tailor pathways for post-extubation transition and rapidly allocate intensive care unit resources without sacrificing the quality of palliative care in the era of COVID-19.

*Trial registration* They study was retrospectively registered. IRB No.: 202101929B0.

**Supplementary Information:**

The online version contains supplementary material available at 10.1186/s40001-022-00972-w.

## Introduction

“Hospice Palliative Care Regulations” were established in Taiwan in 2000, and further amendments on January 10, 2011 stated that terminally ill patients can be extubated. Family meetings are conducted to rule out possible alternative treatment options, determine the irreversibility of the patient’s clinical condition, and reach consensus on the indications for palliative extubation. In cases of withdrawal of life-sustaining treatment (WLST) for unconscious intubated terminal patients who are unable to express their wishes, the appointed medical agent can sign termination consent. Ventilator support can be discontinued to avoid costly and nonbeneficial treatments after a multidisciplinary meeting approved by the hospital’s medical ethics committee. Taiwan pioneers legislation to protect natural death, and promote “advance care planning” and “shared decision-making” [1].

The “Patient Right to Autonomy Act”, the first patient-centered bill in Asia that fully respects a patient’s autonomy, was implemented in 2019. The Act clearly states that everyone has the right to know, choose and make personal medical decisions. For those who make advance directive decisions, are too ill to make decisions, or fall into a coma, their free will is protected and enforced by law. However, the nature of hospice medicine faces challenges from COVID-19. After the first confirmed case of COVID-19 in Taiwan in January 2020, both personal protective equipment and critical care resources have been impacted by the pandemic. The outbreak has severely impacted the daily practice of public health and palliative care globally, and consequently a balance should be struck between intensive care unit (ICU) utilization and hospice medication [2].

Previous articles have focused on heterogeneous factors and prediction models for WLST in different populations, especially with regard to 1-h death for ischemic time of organ donation after cardiac death (DCD). However, a more generalizable tool is needed to evaluate the potential for donation or end-of-life care across ICUs and identify appropriate time points [3]. The Acute Physiology and Chronic Health Assessment (APACHE) II score system is used to assess the severity of critical illness and risk of mortality, and it has been used extensively in the ICU for more than 30 years [4, 5]. It has been validated as a predictor of survival time and mortality in many studies of neurocritical patients, those with terminal diseases, and clinical purposes [6–11].

During the era of the COVID-19 pandemic, the availability of ICU beds is an important issue. The optimal usage of ventilators is of particular importance for COVID-19 critical care. The more known about survival time after terminal extubation can assist in the more efficient use of ICU resources. Current survival models for WLST involve multiple variables and are complex [12]. Therefore, the aim of this study was to develop a simpler model using APACHE II score, which already incorporates many 1-h mortality factors, as ICU staff time is also an important asset in the COVID-19 era. Furthermore, predictors for long-term survival (> 24 h after WLST) are still lacking. Therefore, we developed a 1-day survival model after terminal extubation for critically ill ICU patients.

## Methods

### Study population and setting

The data for this study were obtained from interdisciplinary palliative care team at Chang Gung Memorial Hospital in Keelung and Lovers’ Lake Branch, before and after palliative extubation from 2015 to 2020. All participants were terminally ill, defined as having end-of-life status and no chance of returning to a meaningful life based on the judgment of at least two specialist physicians. Eight patients and/or their families who refused to forgo life-sustaining therapies or withdraw mechanical ventilation were excluded. The terminal extubation process, consistent with the Hospice Palliative Care Act (Natural Death Act) Amendment, was initiated by the families or intensivists after consensus with the family and other medical staff. This retrospective 6-year study was approved by the Institutional Review Board of Chang Gung Medical Foundation Institution, and the requirement for participants’ informed consent was waived (IRB file No. 202101929B0).

### Variables and measures

Different clinical and demographic characteristics were summarized according to extubation status. All possible physiologic and respiratory parameters associated with survival time were calculated. Continuous data are expressed as mean ± standard deviation (SD) or median and interquartile ranges (IQR), and categorical variables are expressed as proportions. Vital signs and oxygen saturation were recorded at the discontinuation of mechanical ventilation. The APACHE II score was reassessed according to the latest laboratory data and physiological variables at extubation. Any unknown or out-of-date variables were scored as 0 points when calculating the APACHE II score.

### Statistical analysis

Continuous variables were analyzed using the Mann–Whitney *U* test, with comparisons of medians for variables with skewed distribution. Categorical variables were compared using Pearson’s Chi-square test or Fisher’s exact test at survival times of 1 h and 24 h. We conducted Cox proportional hazards regression analysis to compute the hazard ratios (HRs) and 95% confidence intervals (CIs) to identify the independent predictors associated with death within 1 h and survival beyond 1 day. Two multiple linear regression models with backward stepwise elimination were conducted on all factors with a *p*-value ≤ 0.2 in univariate analysis. A two-sided *p*-value < 0.05 was considered to indicate statistical significance. Data analyses were performed using SPSS version 26.0 (IBM Corp., Armonk, NY).

## Results

**Fig. 1:**
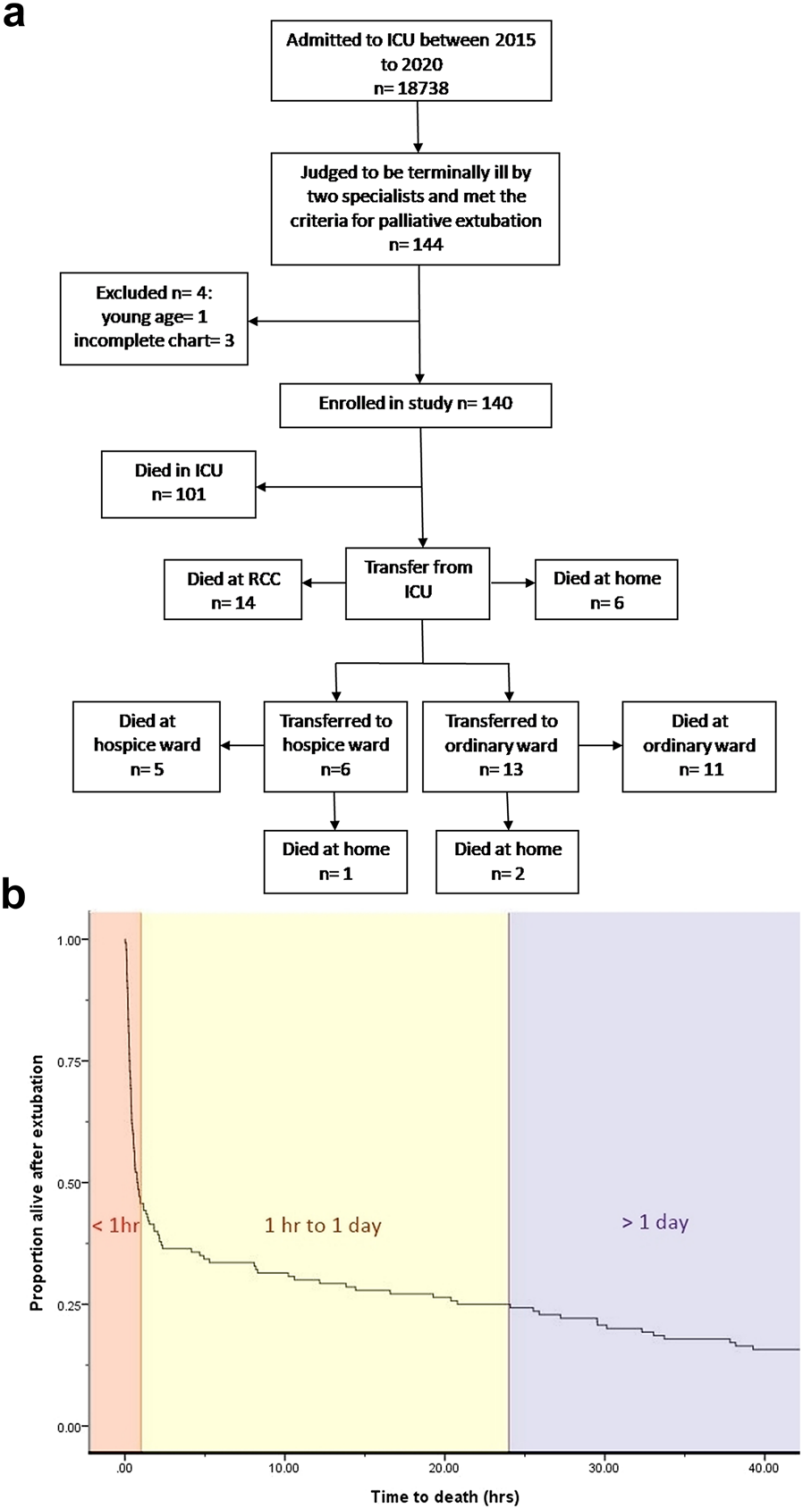
**a** Flow diagram of patient inclusion, exclusion and distribution. **b** Kaplan–Meier estimate of survival after palliative extubation

From January 2015 to December 2020, 18,738 patients who were admitted to an ICU were screened, of whom 144 met the criteria for palliative extubation. Four patients were excluded due to a young age (< 18 years old) or incomplete data (Fig. 1A). The remaining 140 patients who died after palliative extubation after a mean 17.8 days on ventilation (range 1–65 days) were included in the study. Of these patients, 112 (80.0%) had do not resuscitate (DNR) orders prior to family meetings.

### Baseline demographics

The mean age of the study population was 67 years (range 19–94 years), and there were more males (62.1%) than females. Eighty-nine (63.6%) patients were treated with surgical services. Cerebrovascular accident was the main comorbidity (55.0%), followed by congestive heart failure (54.3%), renal failure (45.7%), chronic respiratory disease (42.1%), diabetes mellitus (40.7%), advanced malignancy (37.9%), and chronic liver disease (30.7%). The mean APACHE II score assessed at ICU admission was 25.2 (range 6–42), which increased to 31.3 (range 14–66) at extubation. The clinical characteristics are listed in Table 1.

**Table 1:** Biosociodemographic characteristics in our participants (*n* = 140)

Variables	Mean or number	(SD)	(%)
Baseline characteristics			
Age (years)	66.92	(14.08)	
Gender: male, *n* (%)	87		62.1
Education: more than high school, *n* (%)	58		41.4
Service: surgical, *n* (%)	89		63.6
DNR documented before family meeting, *n* (%)	112		80.0
Donor consideration, *n* (%)	8		5.7
Hospitalization (days)	23.95	(17.29)	
ICU stay (days)	18.64	(14.01)	
Intubation (days)	17.84	(13.63)	
Intubation to family meeting (days)	16.10	(13.15)	
Comorbidities			
Cerebrovascular disease, *n* (%)	83		59.3
Chronic respiratory disease, *n* (%)	59		42.1
Diabetes mellitus, *n* (%)	57		40.7
Heart failure, *n* (%)	76		54.3
Liver disease, *n* (%)	43		30.7
Advanced malignancy, *n* (%)	53		37.9
Renal failure, *n* (%)	64		45.7
Admission category			
Neurology, *n* (%)	40		28.6
Oncology, *n* (%)	47		33.6
Cardiology, *n* (%)	12		8.6
Nephrology, *n* (%)	3		2.1
Chest, *n* (%)	25		17.9
Infection, *n* (%)	13		9.3
Vital signs from monitor			
SBP (mmHg)	102.43	(29.81)	
DBP (mmHg)	56.86	(17.53)	
Mean blood pressure (mmHg)	70.71	(21.63)	
Pulse rate (/min)	88.26	(26.39)	
Respiratory rate (/min)	17.57	(9.04)	
Pulse oximeter (%)	90.05	(15.01)	
Physical variables			
Feeding within 24 h, *n* (%)	110		78.6
Hemodialysis within 3 days, *n* (%)	20		14.3
Presence of IABP, *n* (%)	6		4.3
Tracheostomy, *n* (%)	10		7.1
Neurological variables			
Coma scale (total GCS)	5	(2)	
Motor response (extensor or absent), *n* (%)	70		50.0
Absent Light reflex, *n* (%)	70		50.0
Absent corneal reflex, *n* (%)	73		52.1
Absent cough reflex, *n* (%)	80		57.1
Respiratory variables			
FiO2 from ventilator (%)	48.59	(28.20)	
PEEP (cmH2O)	6.82	(2.15)	
Static pressure (cmH2O)	24.41	(6.97)	
Minute ventilation (L/min)	8.63	(3.74)	
Mean airway pressure (mmHg)	11.92	(3.74)	
Spontaneous breathing trail within 24 h, *n* (%)	57		40.7
Medications previous to withdrawal			
Total dose of opioids within 24 h (mg)	13.33	(21.01)	
Total dose of BZDs within 24 h (mg)	34.00	(93.30)	
Total dose of propofol within 24 h (mg)	80.75	(389.54)	
Opioids use within 24 h, *n* (%)	101		72.1
BZDs use within 24 h, *n* (%)	80		57.1
Propofol use within 24 h, *n* (%)	28		20.0
Inotropic agents use within 24 h, *n* (%)	25		17.9
APACHE II score at ICU admission	25.21	(7.74)	
APACHE II score at extubation	30.58	(8.26)	
Survival time after Extubation (h)	21.83	(53.61)	
Survival more than 1 h (%)	64		45.7
Survival more than 24 h	35		25.0

*SD* standard deviation

### Time to death

The Kaplan–Meier curve for time to death in the 140 extubation patients is shown in Fig, 1B. The time to death after the extubation ranged from 0.02 to 401.72 h (median 0.79 h). Seventy-six patients (54.3%) died within 1 h, and 35 patients (25%) survived beyond 24 h. After extubation, most patients died in the ICU (72.1%), while others died in the ward, hospice and home according to individual circumstances. The mean ICU stay was 18.6 days (range 1–65 days). Of the eight patients (5.7%) whose family members were willing to donate organs, two (1.4%) eventually completed organ transplantation (Additional file 1).

### Univariate analysis

The results of univariate analysis of continuous variables are listed in Table 2. The significant factors (*p*-value < 0.05) associated with death at 1 h of extubation were total Glasgow Coma Scale (GCS) score, diastolic blood pressure (DBP), mean arterial pressure (MAP), pulse rate, respiratory rate, fraction of inspired oxygen (FiO2) from the ventilator, positive end-expiratory pressure (PEEP), static pressure, minute ventilation (MV), and APACHE II score at extubation. The significant factors associated with death at 1 day were GCS score, systolic blood pressure (SBP), DBP, MAP, peripheral arterial oxygen saturation (SpO2) from pulse oximetry, FiO2 from the ventilator, PEEP, static pressure, and MV.

**Table 2:** Univariate analysis of continuous variables at 1 h and 1 day after extubation

	Time to death < 1 h (76)			Time to death ≧ 1 h (64)			*p*^*a*^	Time to death < 1 day (105)			Time to death ≧1 day (35)			*p*^*a*^
	Median	IQR	Min–max	Median	IQR	Min–max		Median	IQR	Min–max	Median	IQR	Min–max	
Baseline characteristics														
Age (years)	64.5	20	19–94	68	20.8	35–93	0.343	67	20.5	19–94	66	20	37–91	0.922
Intubation (days)	14	13	0–65	16	15	1–62	0.156	14	13	0–65	17	16	3–62	0.116
Intubation to family meeting (days)	13	12	0–62	14	15.8	1–62	0.412	13	12	0–62	14	17	1–62	0.329
GCS (total)	3	2.8	3–10	6	2	3–11	< 0.001	3	3	3–1	6	4	3–9	0.040
Vital signs from monitor														
SBP (mmHg)	98.5	40.5	35–165	111	41.5	53–179	0.179	101	39.0	35–169	116	54.0	61–179	0.040
DBP (mmHg)	51	21.0	12–110	57	27	39–105	0.029	51	19.0	12–110	67	25.0	43–97	< 0.001
MAP (mmHg)	66	28.8	0–128.3	74.3	30.4	44–125.3	0.029	66	26.0	0–128.3	83	34.3	56–111.7	0.011
Pulse rate (/min)	84	47.5	26–155	97	23.8	50–164	0.007	89	43.0	26–164	96	23.0	57–126	0.205
Pulse oximetry (SpO2, %)	95	16	0–100	97	4	55–100	0.109	95	13	0–100	97	4	57–100	0.008
Respiratory variables														
Respiratory rate (/min)	15	9.8	0–50	20	8	0–45	< 0.001	16	10.5	0–50	18	8.0	11–45	0.241
FiO2 from ventilator (%)	60	65	21–100	30	5	21–100	< 0.001	45	55	21–100	25	9	21–45	< 0.001
PEEP (cmH2O)	8	3	5–14	5	3	5–13	0.003	8	3	5–14	5	1	5–8	< 0.001
Static pressure (cmH2O)	26	9.0	5–43	22	8.8	7–41	0.011	25	9.0	5–43	21	9.0	7–31	0.019
Minute ventilation (L/min)	10.3	4.5	3.5–19.6	5.9	2.3	2.1–16.3	< 0.001	9.29	4.9	3.5–19.6	5.2	2.3	2.1–16.3	< 0.001
Medications prior to extubation														
Dose of opioids within 24 h (mg)	10	10	0–146	10	15	0–90	0.077	10	10	0–146	10	10	0–90	0.913
Dose of BZDs within 24 h (mg)	1.8	5	0–382.50	5	13.48	0–684.50	0.050	8	7	0–684.50	5	10	0–348.20	0.823
Dose of propofol within 24 h (mg)	0	0	0–4400	0	0	0–680	0.899	0	0	0–4400	0	16.67	0–680	0.464
APACHE II score														
At ICU admission	26	10	6–41	24.5	11.25	6–43	0.723	25	10	6–41	23	11	6–40	0.283
At extubation	32	10.25	19–66	23	9.25	14–40	< 0.001	29	10	17–66	23	8	14–37	0.172

*IQR* interquartile range, *Min* minimum, *Max* maximum

^*a*^*p* values were calculated from Mann–Whitney *U* test comparison of medians, bold = *p*-value < 0.05

The results of univariate analysis of categorical variables are listed in Table 3. The significant factors associated with death at 1 h of extubation were comorbid cerebrovascular disease, chronic respiratory disease, advanced malignancy, SpO2 ≥ 96%, MV ≥ 12 L/min, spontaneous breathing trial (SBT) within the past 24 h, use of inotropic agents in the past 12 h, and APACHE II score at extubation ≥ 25 and ≥ 30. The significant factors associated with death at 1 day were SpO2 ≥ 96%, MV ≥ 12 L/min, SBT within the past 24 h, and the use of inotropic agents in the past 12 h.

**Table 3:** Univariate analysis of categorical variables at 1 h and 1 day after extubation

	Time to death < 1 h (76)		Time to death ≧ 1 h (64)		Statistics		Time to death < 1 d (105)		Time to death ≧ 1 d (35)		Statistics	
	*n*	*%*	*n*	*%*	*χ*2	*p*^*a*^	*n*	*%*	*n*	*%*	*χ*2	*p*^*a*^
Demographics												
Gender (male)	44	50.6	43	49.4	1.275	0.259	63	72.4	24	27.6	0.820	0.365
Education (more than high school)	76	54.3	64	45.7	2.417	0.120	105	75.0	35	25.0	0.039	0.843
Medical service	46	51.7	43	48.3	0.666	0.415	65	73.0	24	27.0	0.504	0.478
Surgical service	30	58.8	21	41.2	0.666	0.415	40	78.4	11	21.6	0.504	0.478
DNR signed before family meeting	76	54.3	64	45.7	0.259	0.611	105	75.0	35	25.0	2.143	0.143
Comorbidities												
Cerebrovascular disease	51	66.2	26	33.8	9.843	0.002	58	75.3	19	24.7	0.010	0.922
Chronic respiratory disease	26	44.1	33	55.9	4.290	0.038	45	76.3	14	23.7	0.088	0.767
Diabetes	32	56.1	25	43.9	0.133	0.751	40	70.2	17	29.8	1.194	0.275
Congestive heart failure	40	52.6	36	47.4	0.183	0.669	55	72.4	21	27.6	0.614	0.433
Chronic liver disease	23	53.5	20	46.5	0.016	0.900	32	74.4	11	25.6	0.011	0.916
Advanced malignancy	22	41.5	31	58.5	5.610	0.018	39	73.6	14	26.4	0.091	0.763
Renal failure	37	57.8	27	42.2	0.591	0.442	41	73.4	17	26.6	0.154	0.695
Physiological variables												
Organ donor consideration	4	50.0	4	50.0	0.063	1.000	5	62.5	3	37.5	0.707	0.412
Nutrition within 24 h	57	51.8	53	48.2	1.259	0.262	81	73.6	29	26.4	0.509	0.476
Dialysis within 3 days	12	60.0	8	40.0	0.307	0.580	15	75.0	5	25.0	0.000	1.000
Presence of IABP	4	66.7	2	33.3	0.387	0.668	5	83.3	1	16.7	0.232	1.000
SpO2 ≥ 96 (%)	35	46.1	41	53.9	4.541	0.033	50	65.8	26	34.2	7.522	0.006
SpO2 ≥ 99 (%)	16	59.3	11	40.7	0.333	0.567	18	66.7	9	33.3	1.239	0.266
Respiratory variables												
Tracheostomy	4	40.0	6	60.0	0.886	0.512	7	70.0	3	30.0	0.144	0.711
Minute ventilation ≥ 12 (L/min)	53	46.5	61	53.5	15.028	< 0.001	81	71.7	33	28.9	5.101	0.024
Spontaneous breathing trail in 24 h	5	8.8	52	91.2	80.255	< 0.001	24	42.1	33	57.9	55.485	< 0.001
Medications prior to extubation												
Opioids use in 24 h	54	54.8	47	46.2	0.098	0.754	76	75.2	25	24.8	0.012	0.913
BZD use in 24 h	38	47.5	42	52.5	3.464	0.063	60	75.0	20	25.0	0.000	1.000
Propofol use in 24 h	15	53.6	13	46.4	0.007	0.932	19	67.9	9	32.1	0.952	0.329
Inotropic agents use in 12 h	21	84.0	4	16.0	10.828	0.001	25	100.0	0	0.0	10.145	0.001
APACHE II score at extubation												
≥ 25	74	73.3	27	26.7	52.640	< 0.001	80	79.2	21	20.8	3.424	0.064
≥ 30	55	80.9	13	19.1	37.690	< 0.001	55	80.9	13	19.1	2.440	0.118

^*a*^*p* values were calculated from the Pearson’s Chi-square test, bold = *p*-value < 0.05

### Multivariate analysis

The results of Cox proportional hazards regression analysis for survival at 1 h and 1 day are shown in Table 4. In the 1-h model, intubation duration, SpO2 from pulse oximetry, total dose of opioids within 24 h, total dose of BZDs within 24 h, education level, comorbid cerebrovascular accident had *p*-values ≤ 0.2 in the univariate analysis and were entered into the multivariate analysis. Items that overlapped including GCS, DBP, MAP, pulse rate, respiratory rate, FiO2 from the ventilator in APACHE II score were excluded. To increase clinical relevance, we used MV 12 L/min in regression multivariate analysis according to a previous study [13]. In the 1-day model, intubation duration, APACHE II score at extubation, and DNR orders signed before family meeting had *p*-values ≤ 0.2 in the univariate analysis and were included in the multivariate analysis. Items correlated with APACHE II score were removed. To enhance application, SpO2 < 96% was used in the analysis.

**Table 4:** Multivariate Cox regression analysis of factors associated with patient death within 1 h and survival beyond 1 day

Death within 1 h	RC	HR (95% CI)	*p* value
Intubation (days)	0.010	1.01 (0.99–1.03)	0.219
Pulse oximetry (SpO2) (%)	− 0.006	0.99 (0.98–1.01)	0.468
PEEP (cmH2O)	0.099	1.10 (0.98–1.24)	0.099
Static pressure (cmH2O)	0.035	1.04 (0.99–1.08)	0.093
Total dose of opioids within 24 h (mg)	0.000	1.00 (0.99–1.01)	0.981
Total dose of BZDs within 24 h (mg)	0.001	1.00 (1.00–1.01)	0.519
Education (elementary school or uneducated vs. more than high school)	− 0.432	0.65 (0.39–1.08)	0.095
Cerebrovascular disease (yes vs. no)	0.291	1.34 (0.85–1.58)	0.356
Spontaneous breathing trail in 24 h (no vs. yes)	2.448	11.57 (4.30–31.15)	** < 0.001**
Minute ventilation 12(L/min (≥ 12 vs. < 12)	1.488	4.43 (2.30–8.52)	** < 0.001**
Inotropic agents use in 12 h (yes vs. no)	0.393	1.48 (0.85–2.59)	0.167
APACHE II score 25 (≥ 25 vs. < 25)	2.681	14.60 (3.29–64.78)	** < 0.001**
Survival beyond 1 day			
Intubation (days)	0.004	1.00 (0.99–1.02)	0.557
PEEP (cmH2O)	0.068	1.07 (0.97–1.18)	0.170
Static pressure (cmH2O)	0.027	1.03 (1.00–1.06)	0.092
Minute ventilation (L/min)	0.211	1.24 (1.16–1.32)	** < 0.001**
APACH II score at extubation	0.007	1.01 (0.98–1.04)	0.638
DNR signed before family meeting (yes vs. no)	− 0.012	0.99 (0.76–1.29)	0.928
Pulse oximetry (SpO2 ≥ 96% vs. SpO2 < 96%)	0.264	1.30 (1.05–1.61)	**0.015**
Spontaneous breathing trail in 24 h (yes vs. no)	1.934	6.92 (3.60–13.29)	** < 0.001**
Inotropic agents use in 12 h (no vs. yes)	0.227	1.26 (0.76–2.06)	0.371

*RC* regression coefficient*, HR* hazard ratio, *CI* confidence interval

Bold = *p*-value < 0.05

The final Cox regression model for death within 1 h showed that no SBT within the past 24 h, MV ≥ 12 L/min, and APACHE II score ≥ 25 were associated with higher mortality. Meanwhile, the model for survival beyond 1 day indicated that lower MV, SpO2 ≥ 96%, and SBT within the past 24 h were associated with longer survival.

## Discussion

To the best of our knowledge, this is the first study to use APACHE II score to predict the time to death after terminal extubation. In the 140 terminal patients enrolled in this study, a reassessed APACHE II score 25 at terminal extubation was a practical and helpful tool to assess survival, which may be of particular use in the battle against COVID-19. We also found that no SBT within the past 24 h and MV ≥ 12 L/min were significantly associated with 1-h mortality. As these patients survived for longer than 1 h, APACHE II score was not suitable to predict survival longer than 24 h. SpO2 ≥ 96%, MV, and SBT within the past 24 h could be used as an indication of when to transfer patients from an ICU to hospice unit.

### Reassessed APACHE II score at terminal extubation

**Fig. 2:**
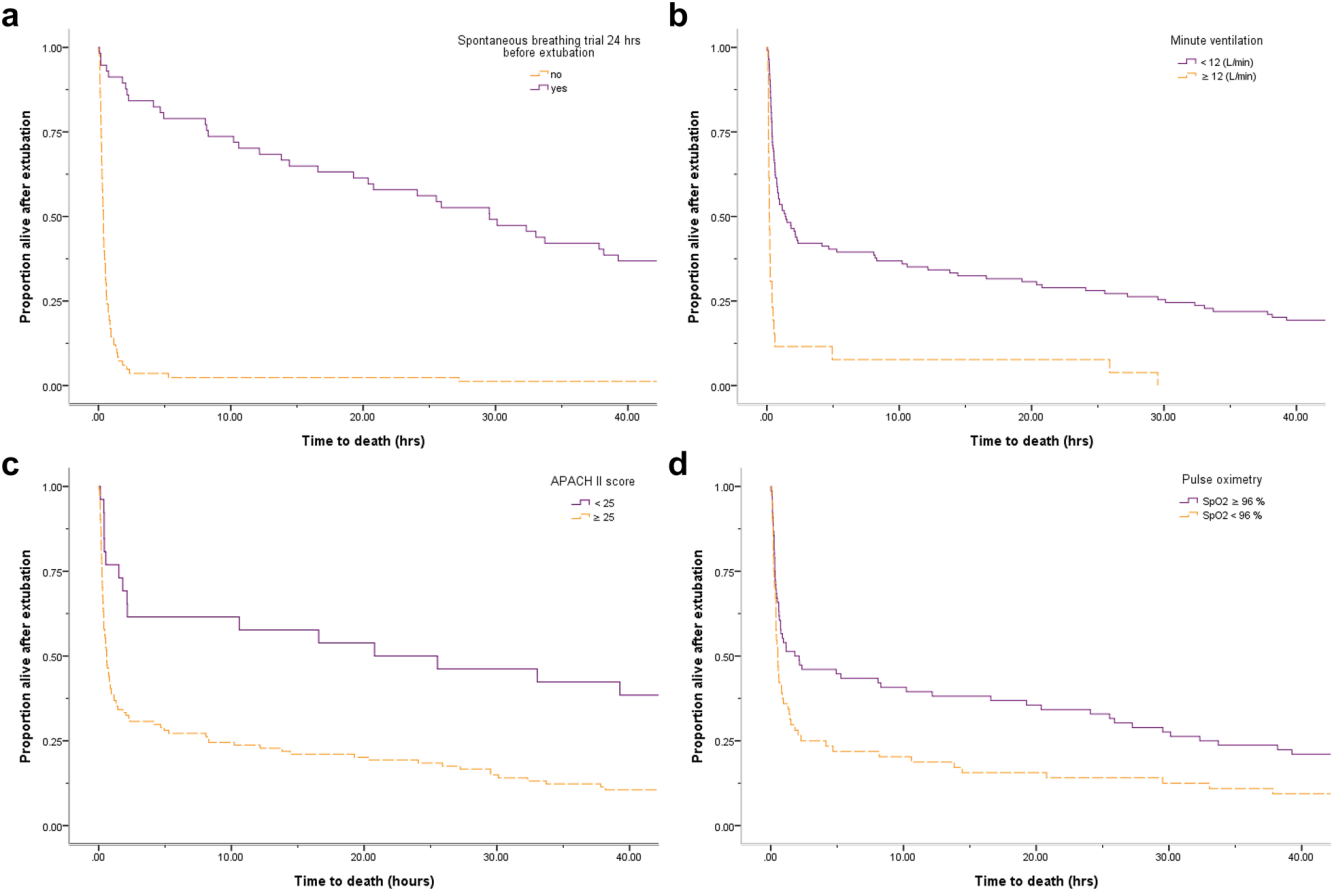
Survival curves by **a** spontaneous breathing trial within the past 24 h, **b** minute ventilation 12 L/min, **c** APACHE II score 25 at extubation, and **d** pulse oximetry: SpO2 96%

Previous studies have reported that various factors are associated with time to death within 1 h after WLST, including age, FiO2, body temperature, MAP, blood pH, heart rate, respiratory rate, serum sodium, potassium, creatinine, white blood cell count, GCS and severe organ system insufficiency, which are also the main variables used to calculate the APACHE II score [3, 6, 12–18]. The APACHE II scoring system is a simple and widely used reproducible ICU prognostic model, and our data showed that it could be used to predict survival time after compassionate extubation. Recalculation of the “Acute Physiology Score” part based on the updated status of the patient after extubation is relatively convenient for multidisciplinary teams. Although the reassessed APACHE II score did not reach significance in the 1-day model, an APACHE II score ≥ 25 closely predicted 1-h mortality (Fig. 2C). As an APACHE II score of 25 represents an approximately 50% mortality rate in clinical practice, a cutoff value of 25 has been well validated in predicting mortality in ventilator-associated pneumonia, emergency surgical patients, and patients with severe sepsis, carbon monoxide poisoning, and hematological cancer [7, 19–21].

Our results are also consistent with discharge APACHE II score being superior to admission APACHE II score in predicting post-ICU mortality [22]. Given that more patient parameters and surgical status are taken into account in the APACHE II score, it can serve as a new prognostic tool for hospice care.

### Respiratory-related factors

Respiratory variables are consistently associated with time to death [13, 15]. With regard to the items excluded from the APACHE II scoring system, MV was of greater significance in multivariate analysis compared with static pressure and PEEP. MV has also been shown to play an important role in predicting noninvasive ventilation failure in patients with early mild acute respiratory distress syndrome (ARDS) induced by pneumonia, successful extubation, and mortality caused by ARDS in patients with COVID-19 [23–25].

SBT, an indicator for liberation from ventilation in different populations, is performed using T-piece ventilation and pressure support ventilation lasting between 0.5 and 2 h [26, 27]. Patients eligible for SBT are screened according to low FiO2 (< 0.5) and PEEP (< 5–8 cmH2O) requirements, stable hemodynamics, and the ability to initiate spontaneous breathing, all of which are also favorable predictors for longer survival after WLST [3, 18, 28]. In addition, the components of the SBT can be used to measure the burden of post-extubation symptoms and guide the anticipatory dose of medication after terminal extubation [29]. It is therefore reasonable that the subjects with lower MV and attempting SPT within 24 h had less dependency on mechanical ventilation and a longer survival (Fig. 2A, B).

### Peripheral arterial oxygen saturation

Pulse oximetry is used to measure SpO2, and pulse oximeters are standard equipment in ICUs [30]. It is considered to be the “fifth vital sign” to monitor systemic oxygen delivery in a noninvasive and continuous fashion, especially in critically ill patients supported by extracorporeal membrane oxygenation and mechanical ventilation [31–33]. A lower SpO2, compared with SpO2 ≥ 96%, has been associated with an increased risk of all-cause mortality in the general adult population [34]. In a systematic review conducted in 2018, oxygen therapy was associated with increased mortality in acutely ill adults with SpO2 > 96% [35]. The authors suggested that critically ill patients with SpO2 ≥ 96% have a lower oxygen demand and better compensation. Our study supports these findings, as SpO2 was the only factor significantly associated with 1-day survival (Fig. 2D). Moreover, the target of SpO_2_ should be 92% to 96% in adults with COVID-19 who need supplemental oxygen according to treatment guidelines as home pulse oximetry has become increasingly popular during the COVID-19 era [36].

### Disposition after palliative extubation in the COVID-19 pandemic era

Our results showed that combining the APACHE II score with other respiratory parameters was effective in predicting 1-h mortality. Therefore, our model could be used to identify which terminal patients with irreversible illness should remain in the ICU without being transferred, regardless of comorbidities. Our 1-h model could help physicians to quickly detect suitable candidates for DCD. The 1-day model based on SpO2 could be used to identify patients with a likelihood of longer survival, as a significant minority are discharged alive after palliative extubation [5, 37]. Transition to a general care ward, hospice department ward or home where comfort-oriented care can be provided is suitable for patients who are predicted to survive for more than 1 day according to the consensus of family meetings [12] (Fig. 1B).

ICU facilities are important for patients with moderate and severe COVID-19 infection. When COVID-19 peaks occur, hospitals may run out of beds and other medical supplies. In this situation, available ICU beds are recruited by the government to avoid collapse of the health system. Hospital capacity is consequently reduced, and other medical practices including hospice care are also likely to be over-utilized. Under the “coexisting with the virus” and “zero severe cases” policy of the Ministry of Health and Welfare in Taiwan, subjects who are expected to survive for 1 h to 1 day can be transferred to palliative home care directly depending on religious needs and individual differences. Some studies have also suggested that ICU specialist opinion was closely associated with the time of death [16]. When staff are overwhelmed by the number of COVID patients, a simple global guide is needed to avoid overloading healthcare systems and the guilt that decision-making can create.

Furthermore, DNR orders, consideration of organ donation, and hospice medications for pain/symptom relief did not affect the time to natural death in the multivariate analysis. Adequate medications and supplies should be considered based on probable survival time to reduce distress and family anxiety during and after transfer from the ICU. Although not a perfect substitute, well-designed apps and online counseling are practical in home hospice practice [38].

## Limitations and strengths

As this was a retrospective study, some data were not re-examined at the time of compassionate extubation to reduce possible patient discomfort. In addition, we enrolled terminally ill Asian patients from one institute, and the sample size was relatively small. Further external validation studies are needed. Nonetheless, reassessed APACHE II score compensated for the missing lab data, and we provide a convenient tool with the potential for global use to avoid the interference of repeated testing during natural death. The 1-day model offers evidence for dealing with contradictions between COVID-19 treatment and hospice care. Data on survival time after WSLT in Asia are extremely limited due to legal and religious restrictions, so our results add to the knowledge of this group of patients. Further artificial intelligence analysis of different studies could be used to prospectively validate models applied to other ICUs and decision-making after extubation [12, 38].

## Conclusions

In conclusion, the accurate estimation of time to death can optimize the use of hospital resources. The 1-h and 1-day models showed that a reassessed APACHE II score of ≥ 25 and SpO2 ≥ 96%, respectively, were practical predictors of mortality in the terminal patients in this study. These clinical factors may help to objectively tailor pathways for post-extubation transition and rapidly allocate ICU resources without sacrificing the quality of palliative care in the era of COVID-19. (Additional file1: Fig S1)

## Supplementary Information

**Additional file 1: Fig S1.** Trends in ICUs utilization and length of stay.

## Data Availability

The datasets generated during and/or analyzed during the current study are available from the corresponding author on reasonable request.

## Abbreviations

APACHE: Acute Physiology and Chronic Health Evaluation
ARDS: Acute respiratory distress syndrome
BZD: Benzodiazepines
CI: Confidence interval
CNS: Central nervous system
CVA: Cerebrovascular accident
DBP: Diastolic blood pressure
DCD: Donation after cardiac death
DNR: Do no resuscitation
ECMO: Extracorporeal membrane oxygenation
FiO2: Fraction of inspired oxygen
GCS: Glasgow Coma Scale
HR: Hazard ratio
ICU: Intensive care unit
IQR: Interquartile range
MAP: Mean arterial pressure
OS: Overall survival
PEEP: Positive end-expiratory pressure
RC: Regression coefficient
SBP: Systolic blood pressure
SBT: Spontaneous breathing trial
SD: Standard deviation
SpO2: Peripheral arterial oxygen saturation
WLST: Withdrawal of life-sustaining therapy

## References

[1] Hung YS, Lee SH, Hung CY, Wang CH, Kao CY, Wang HM, Chou WC (2018). Clinical characteristics and survival outcomes of terminally ill patients undergoing withdrawal of mechanical ventilation. J Formos Med Assoc.

[2] Ruangsomboon O, Boonmee P, Nimmannit A (2021). The COVID-19 pandemic: the effect on airway management in non-COVID emergency patients. BMC Emerg Med.

[3] Munshi L, Dhanani S, Shemie SD, Hornby L, Gore G, Shahin J (2015). Predicting time to death after withdrawal of life-sustaining therapy. Intensive Care Med.

[4] Barie PS, Hydo LJ, Fischer E (1995). Comparison of APACHE II and III scoring systems for mortality prediction in critical surgical illness. Arch Surg.

[5] Knaus WA, Draper EA, Wagner DP, Zimmerman JE (1985). APACHE II: a severity of disease classification system. Crit Care Med.

[6] Kuo WK, Hua CC, Yu CC, Liu YC, Huang CY (2020). The cancer control status and APACHE II score are prognostic factors for critically ill patients with cancer and sepsis. J Formos Med Assoc.

[7] Koperna T, Semmler D, Marian F (2001). Risk stratification in emergency surgical patients: is the APACHE II score a reliable marker of physiological impairment?. Arch Surg.

[8] Chawla LS, Seneff MG, Nelson DR, Williams M, Levy H, Kimmel PL, Macias WL (2007). Elevated plasma concentrations of IL-6 and elevated APACHE II score predict acute kidney injury in patients with severe sepsis. Clin J Am Soc Nephrol.

[9] Raj R, Siironen J, Kivisaari R, Hernesniemi J, Skrifvars MB (2014). Predicting outcome after traumatic brain injury: development of prognostic scores based on the IMPACT and the APACHE II. J Neurotrauma.

[10] Rowe M, Brown J, Marsh A, Thompson J (2022). Predicting mortality following traumatic brain injury or subarachnoid hemorrhage: an analysis of the validity of standardized mortality ratios obtained from the APACHE II and ICNARCH-2018 models. J Neurosurg Anesthesiol.

[11] Godino M, Tommasino N, Mizraji R, Carámbula A, Cacciatori A, Leyes L, Bengochea M (2020). Prediction of the evolution to brain death in the neurocritical patient: SPN model showed better performance than simplified acute physiology score II and acute physiology and chronic health evaluation II.

[12] Winter MC, Day TE, Ledbetter DR, Aczon MD, Newth CJL, Wetzel RC, Ross PA (2021). Machine learning to predict cardiac death within 1 hour after terminal extubation. Pediatr Crit Care Med.

[13] Huynh TN, Walling AM, Le TX, Kleerup EC, Liu H, Wenger NS (2013). Factors associated with palliative withdrawal of mechanical ventilation and time to death after withdrawal. J Palliat Med.

[14] Kotsopoulos AMM, Boing-Messing F, Jansen NE, Vos P, Abdo WF (2018). External validation of prediction models for time to death in potential donors after circulatory death. Am J Transplant.

[15] Long AC, Muni S, Treece PD, Engelberg RA, Nielsen EL, Fitzpatrick AL, Curtis JR (2015). Time to death after terminal withdrawal of mechanical ventilation: specific respiratory and physiologic parameters may inform physician predictions. J Palliat Med.

[16] Brieva J, Coleman N, Lacey J, Harrigan P, Lewin TJ, Carter GL (2013). Prediction of death in less than 60 minutes following withdrawal of cardiorespiratory support in ICUs. Crit Care Med.

[17] Yee AH, Rabinstein AA, Thapa P, Mandrekar J, Wijdicks EF (2010). Factors influencing time to death after withdrawal of life support in neurocritical patients. Neurology.

[18] Suntharalingam C, Sharples L, Dudley C, Bradley JA, Watson CJ (2009). Time to cardiac death after withdrawal of life-sustaining treatment in potential organ donors. Am J Transplant.

[19] Zhou XY, Ben SQ, Chen HL, Ni SS (2015). A comparison of APACHE II and CPIS scores for the prediction of 30-day mortality in patients with ventilator-associated pneumonia. Int J Infect Dis.

[20] Mohamed AKS, Mehta AA, James P (2017). Predictors of mortality of severe sepsis among adult patients in the medical intensive care unit. Lung India.

[21] Liao WC, Cheng WC, Wu BR, Chen WC, Chen CY, Chen CH, Tu CY, Hsia TC (2019). Outcome and prognostic factors of patients treated in the intensive care unit for carbon monoxide poisoning. J Formos Med Assoc.

[22] Lee H, Lim CW, Hong HP, Ju JW, Jeon YT, Hwang JW, Park HP (2015). Efficacy of the APACHE II score at ICU discharge in predicting post-ICU mortality and ICU readmission in critically ill surgical patients. Anaesth Intensive Care.

[23] Martinez A, Seymour C, Nam M (2003). Minute ventilation recovery time: a predictor of extubation outcome. Chest.

[24] Fusina F, Albani F, Bertelli M, Cavallo E, Crisci S, Caserta R, Nguyen M, Grazioli M, Schivalocchi V, Rosano A (2021). Corrected minute ventilation is associated with mortality in ARDS caused by COVID-19. Respir Care.

[25] He H, Sun B, Liang L, Li Y, Wang H, Wei L, Li G, Guo S, Duan J, Li Y (2019). A multicenter RCT of noninvasive ventilation in pneumonia-induced early mild acute respiratory distress syndrome. Crit Care.

[26] Subirà C, Hernández G, Vázquez A, Rodríguez-García R, González-Castro A, García C, Rubio O, Ventura L, López A, de la Torre M-C (2019). Effect of pressure support vs T-piece ventilation strategies during spontaneous breathing trials on successful extubation among patients receiving mechanical ventilation: a randomized clinical trial. JAMA.

[27] Ferreira FV, Sugo EK, Aragon DC, Carmona F, Carlotti A (2019). Spontaneous breathing trial for prediction of extubation success in pediatric patients following congenital heart surgery: a randomized controlled trial. Pediatr Crit Care Med.

[28] Davila D, Ciria R, Jassem W, Briceno J, Littlejohn W, Vilca-Melendez H, Srinivasan P, Prachalias A, O'Grady J, Rela M (2012). Prediction models of donor arrest and graft utilization in liver transplantation from maastricht-3 donors after circulatory death. Am J Transplant.

[29] Yeow M-E, Chen E (2020). Ventilator withdrawal in anticipation of death: the simulation lab as an educational tool in palliative medicine. J Pain Symptom Manage.

[30] Van de Louw A, Cracco C, Cerf C, Harf A, Duvaldestin P, Lemaire F, Brochard L (2001). Accuracy of pulse oximetry in the intensive care unit. Intensive Care Med.

[31] Chan ED, Chan MM, Chan MM (2013). Pulse oximetry: understanding its basic principles facilitates appreciation of its limitations. Respir Med.

[32] Jubran A (2004). Pulse oximetry. Intensive Care Med.

[33] van den Boom W, Hoy M, Sankaran J, Liu M, Chahed H, Feng M, See KCJC (2020). The search for optimal oxygen saturation targets in critically ill patients: observational data from large ICU databases. Chest.

[34] Vold ML, Aasebø U, Wilsgaard T, Melbye HJBpm (2015). Low oxygen saturation and mortality in an adult cohort: the tromsø study. BMC Pulm Med.

[35] Chu DK, Kim LH, Young PJ, Zamiri N, Almenawer SA, Jaeschke R, Szczeklik W, Schünemann HJ, Neary JD, Alhazzani WJTL (2018). Mortality and morbidity in acutely ill adults treated with liberal versus conservative oxygen therapy (IOTA): a systematic review and meta-analysis. Lancet.

[36] Shenoy N, Luchtel R, Gulani P (2020). Considerations for target oxygen saturation in COVID-19 patients: are we under-shooting?. BMC Med.

[37] Pan CX, Platis D, Maw MM, Morris J, Pollack S, Kawai F (2016). How long does (s)he have? retrospective analysis of outcomes after palliative extubation in elderly, chronically critically ill patients. Crit Care Med.

[38] Lee PH, Peng JK, Chang HC, Huang PS, Wu CY, Hsu SH, Weng YC, Tu CY, Lee JH, Chiu GL (2021). Strategies maintaining hospice and palliative care quality during COVID-19 pandemic in Taiwan. BMJ Support Palliat Care.

