# Supplementary figures and images for: Prediction of survival time after terminal extubation: the balance between critical care unit utilization and hospice medicine in the COVID-19 pandemic era
Source: Eur J Med Res. 2023 Jan 11;28:21. doi: 10.1186/s40001-022-00972-w (PMC9832251; doi:10.1186/s40001-022-00972-w)

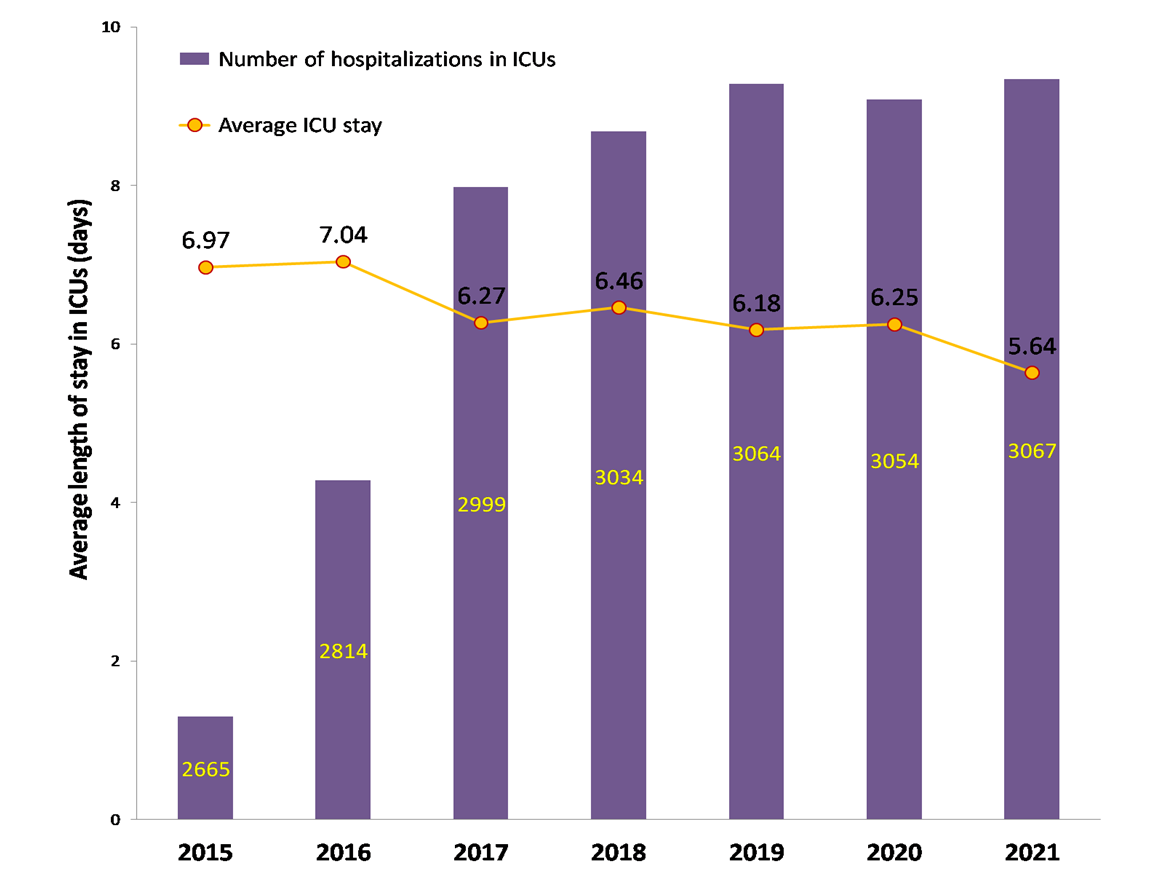

Supplement: Supplementary file 1 — Additional file 1: Fig S1. Trends in ICUs utilization and length of stay. [file 40001_2022_972_MOESM1_ESM.tif]
